# Comparing the performance of statistical, machine learning, and deep learning algorithms to predict time-to-event: A simulation study for conversion to mild cognitive impairment

**DOI:** 10.1371/journal.pone.0297190

**Published:** 2024-01-22

**Authors:** Martina Billichová, Lauren Joyce Coan, Silvester Czanner, Monika Kováčová, Fariba Sharifian, Gabriela Czanner

**Affiliations:** 1 Faculty of Informatics and Information Technologies, Slovak University of Technology in Bratislava, Bratislava, Slovakia; 2 School of Computer Science and Mathematics, Liverpool John Moores University, Liverpool, United Kingdom; Chunghwa Telecom Co. Ltd., TAIWAN

## Abstract

Mild Cognitive Impairment (MCI) is a condition characterized by a decline in cognitive abilities, specifically in memory, language, and attention, that is beyond what is expected due to normal aging. Detection of MCI is crucial for providing appropriate interventions and slowing down the progression of dementia. There are several automated predictive algorithms for prediction using time-to-event data, but it is not clear which is best to predict the time to conversion to MCI. There is also confusion if algorithms with fewer training weights are less accurate. We compared three algorithms, from smaller to large numbers of training weights: a statistical predictive model (Cox proportional hazards model, CoxPH), a machine learning model (Random Survival Forest, RSF), and a deep learning model (DeepSurv). To compare the algorithms under different scenarios, we created a simulated dataset based on the Alzheimer NACC dataset. We found that the CoxPH model was among the best-performing models, in all simulated scenarios. In a larger sample size (n = 6,000), the deep learning algorithm (DeepSurv) exhibited comparable accuracy (73.1%) to the CoxPH model (73%). In the past, ignoring heterogeneity in the CoxPH model led to the conclusion that deep learning methods are superior. We found that when using the CoxPH model with heterogeneity, its accuracy is comparable to that of DeepSurv and RSF. Furthermore, when unobserved heterogeneity is present, such as missing features in the training, all three models showed a similar drop in accuracy. This simulation study suggests that in some applications an algorithm with a smaller number of training weights is not disadvantaged in terms of accuracy. Since algorithms with fewer weights are inherently easier to explain, this study can help artificial intelligence research develop a principled approach to comparing statistical, machine learning, and deep learning algorithms for time-to-event predictions.

## Introduction

### Background

The continuous improvement of lifestyle and healthcare has led to an increase in the average life expectancy worldwide. However, longer life expectancy also increases the likelihood of developing age-related brain disorders, which can have severe impacts on daily life and even be direct causes of death [1]. One such disorder is Alzheimer’s disease (AD), which causes a decline in cognitive abilities such as memory and concentration. These cognitive changes contribute to the loss of the ability to independently perform basic everyday activities. AD causes dementia symptoms that become worse over time and are irreversible. Alzheimer’s patients face the risk of progressing to mild cognitive impairment (MCI) and later to dementia. While dementia currently has no cure, promising therapies can slow down the progression of individuals who are not yet at the MCI stage [2]. Therefore, accurately predicting the time of progression to MCI in cognitively normal patients is of great importance.

In order to accurately predict the time to MCI progression, it is crucial to have access to a well-structured dataset that captures the necessary information. Such a dataset must have a recording of time when a patient progressed to MCI along with other variables, and it is called *survival dataset* [3]. These types of datasets are often also referred to as *time-to-event data*, as the ground truth we measure is the time to occurrence of the event of interest. In our case, the event of interest is the conversion to MCI, which we refer to as the *time to MCI progression*.

A typical feature of survival data is censoring. Censoring refers to situations where the exact occurrence time of an event of interest is unknown for certain subjects in the study [4]. For example, some subjects are lost to follow up, i.e. they left the study for an MCI-unrelated reason such as death, refusal to continue to be in the study, or relocation. Other subjects’ date of conversion to MCI can be unknown because the researcher had to end the study or the data collection before the subject converted.

In electronic health records (also called routinely collected datasets) and national observational studies, such as the National Alzheimer’s Coordinating Center (NACC), data is collected routinely, often up to a specific time point. This practice creates censoring for subjects who are still being followed but have not yet experienced the event of interest, in this case, the progression to MCI. Patients who dropped out of the study, died, or still exhibit normal cognition are considered right censored. It is important to recognize that censored times should not be treated as missing data. Instead, they provide valuable information by indicating that the event has not yet occurred for those individuals. Treating censored times as missing would disregard this useful information. Therefore, any algorithm aiming to predict the time to MCI progression must take into account both the event times and the censoring times.

Various automatic prediction algorithms have been developed and used to predict the time to event, however, only one paper has been published to predict time to MCI progression [5]. Generally, in the medical field, there are three main types of predictive algorithms: (A) classical statistical predictive models, (B) machine learning algorithms and (C) Deep learning algorithms. From classical statistics, there are methods used for survival analysis: Kaplan-Meier estimator for estimating the survival function [6] together with Log-rank test as an approach to assess whether the Kaplan-Meier curves for two (or more) groups are statistically equivalent [3]. Another widely used type of classical statistical model is semiparametric Cox proportional hazard regression model [7], which can be used to study risk factors as well as for prediction (thus belonging to a set of so called statistical predictive models). In addition to classical statistical models, machine learning methods have emerged as powerful tools for time-to-event analysis. Two notable machine learning algorithms used in survival analysis are Random Survival Forest (RSF) and Support Vector Machine (SVM) [8]. Among the deep learning models employed in time-to-event analysis, DeepSurv stands out as one of the most widely utilized methods, utilizing neural networks as its fundamental framework [9].

### Related work

Limited research has been conducted comparing analytical techniques for automatic prediction of time to event in medical applications. However, a recent methodological review by Smith et al. [10] addressed this gap by examining relevant studies. The review identified 10 papers that met the study inclusion criteria. Although none of the studies focused on Alzheimer’s disease, they employed Monte Carlo simulations based on clinical time-to-event data. The Cox proportional hazard model was the most commonly applied method in the studies reviewed and it was compared to machine learning approaches such as Random Survival Forest (RSF), survival Support Vector Machine (SVM), or neural networks. Multiple evaluation metrics, such as the C-index, Integrated Brier score (IBS), and Mean Squared Prognostic Error (MSPE), were utilized to assess the performance of these models. However, the review did not yield a conclusive finding regarding the most accurate technique and its optimal scenario [10].

Clinical studies, particularly those focused on dementia, often involve data that is high-dimensional, censored, and may contain missing information. These complexities pose challenges when predicting future events. While there is currently no cure for dementia, interventions exist that can slow down the progression to cognitive impairment. Therefore, early detection, even before symptoms manifest, is crucial for successful management. Spooner et al. [11] emphasize the need for machine learning models capable of predicting the onset of dementia. In their study, they compared ten machine learning algorithms in conjunction with eight feature selection methods, specifically designed for survival analysis of high-dimensional clinical data. The performance of these models was evaluated using the C-index metric.

Overall, the study conducted by Spooner et al. [11] revealed that the Cox model without feature selection exhibited the poorest performance, with a decline of 7% and 21% in the C-index metric. Conversely, both the Cox model with feature selection and the Random Forest survival models demonstrated higher and comparable performance in predicting dementia onset, based on the analysis of two clinical datasets. When it comes to predicting the conversion to MCI, it is crucial to employ accuracy evaluation metrics such as the C-Index, Integrated Brier Score (IBS), or Mean Squared Prognostic Error (MSPE). In certain medical domains, different algorithms may yield similar levels of accuracy, as demonstrated by Spooner et al. [11].

In a review of predictive algorithms based on cross-sectional studies Christodoulou et al. [12] compared the performance of logistic regression with machine learning methods. Their findings indicated no significant difference in performance between these two approaches. As a result, the authors emphasized the necessity for further studies that directly compare the performance of different predictive algorithms.

When multiple algorithms demonstrate similar accuracy, it provides an opportunity to consider other desirable properties in the decision-making process. For instance, simplicity, explainability, and trustworthiness are important factors to consider. Simplicity refers to an algorithm that uses a smaller number of features or features that are easier to obtain (e.g. non-imaging features), or are more reliable to obtain (e.g. not prone to recall bias), while still achieving comparable accuracy to more complex algorithms. Explainability refers to an algorithm’s ability to provide two types of explanations: 1) explaining how it works, its capabilities, and evaluation, and 2) clarifying why and how it reached a specific conclusion. These explanations should be understandable to a range of users, with the level of detail adjusted to the context. By offering clear explanations, an algorithm promotes transparency, and trust, and enables users to assess its reliability and limitations [13]. Explainable AI can be achieved through two approaches: constructing the model as inherently explainable, like the Cox model, or incorporating explainability features afterward (post-hoc approaches). We consider AI as trustworthy if it demonstrates honesty, competency, and reliability [14, 15]. For example, an AI system is considered trustworthy when it can openly express uncertainty regarding its own claims, demonstrating transparency and integrity. The trustworthiness of AI, including uncertainty-aware AI, is a current area of research and focus as expressed by the European Commission and its High-Level Expert Groups.

The relationship between the accuracy of prediction algorithms and the size of training data is not fully understood due to the complexity of both the data and the algorithms involved. This holds true for both cross-sectional studies (e.g., diagnostic studies) and longitudinal studies (e.g., survival studies). Consequently, there may not be readily available mathematical formulas for determining sample sizes in such cases. In Cox regression, the determination of sample size depends on the specific goal of the analysis, whether it is to assess associations or make predictions. In an association study, we compare groups (younger vs older) with respect to time to event, in prediction studies we predict individual time to event. Predicting individuals is harder than comparing groups, hence we need more data for training if the aim is to predict individuals. If Cox regression is used to learn about associations then there are methods to calculate the sample size [16] which increases with an increasing number of features in Cox regression. If Cox regression is used for the prediction (prognosis) the sample size needs to be even higher, however, this is not fully understood [16]. This gap in knowledge highlights the need for further research in this area. For modern machine learning algorithms like Support Vector Machines (SVM), Random Forest (RF), or deep learning methods, it is generally known that larger sample sizes are necessary to achieve higher accuracy and mitigate the risk of overfitting [17]. This emphasizes the importance of investigating the impact of training sample sizes on the accuracy and precision of predictive algorithms.

It is also not clear how predictive algorithms compare under different survival distributions of data e.g. distribution of time to event. Due to suggestions that machine learning approaches may be able to improve clinical outcome predictions, Austin et al. [18] explored in their paper the predictive performance of machine and statistical learning methods. Their main aim was to identify which machine learning approaches outperform classical statistical learning methods. The study focused on two large simulated cardiovascular datasets with a binary outcome variable indicating whether the patient died within 30 days. Six different methods were evaluated: bagged classification trees, stochastic gradient boosting machines using trees as base learners, random forests and lasso, ridge, and unpenalized logistic regression. Eight performance metrics were employed to assess the performance of these methods. The results of the study indicated that the performance of all methods was similar, with no single method emerging as superior across all the metrics used. However, the authors noted that certain methods, such as random forests, exhibited poorer performance when the outcomes were generated under a model different from random forests. The study was limited, however, as the datasets used were similar in size thus it was recommended that further analysis is required to compare the model’s performance on datasets of varying sizes.

When it comes to prediction problems in human studies there is another challenge: the heterogeneity of patients. For example, Gao et al. [19] described this problem and claimed that heterogeneity is not uncommon among patients in many diseases and ignoring this issue might lead to poor clinical diagnostics. Ignored heterogeneity leads to biases in estimated weights and in inefficient prediction (e.g. analysis of highway accident data [20]). Therefore, it is crucial to consider and address heterogeneity when developing predictive models. Heterogeneity can manifest as observed or unobserved. When differences between two or more groups relate to observable characteristics (e.g. differences in progression risk in males and females relating to cognitive status), this is observed heterogeneity. Such characteristics (gender and cognitive status) can be used to partition the patients into separate groups and carry out group-specific analyses (either separate analyses or one analysis with relevant dummy variables flagging separate groups, called interaction terms in statistics). Unobserved heterogeneity occurs when in addition to the observed variables under study, there exist other relevant variables that are unobserved but correlated with the observed variables (with outcome variables and/or with predictors).

A fair comparison of algorithms should be conducted on the same dataset, which can either be real datasets or simulated datasets. Smith et al. [10], in their review, highlighted the advantages of simulation studies. These studies allow us to understand the performance of methods under known data-generating mechanisms and enable comparisons in various settings, such as varying sample sizes or complexity of relationships between predictors. Simulation studies provide valuable insights into how different methods perform under different scenarios. However, it is important to ensure that the data simulation process closely resembles the characteristics of real data from the clinical domain. By using realistic data simulation techniques, we can gain a better understanding of how predictive algorithms will perform in real-world applications.

In summary, to our knowledge, there is currently no comparison of algorithms focused on predicting the time to conversion to MCI. Important areas to investigate are the effect of sample sizes, the effect of the survival distribution of the data, and the effect of heterogeneity on model performance.

### Aim and objectives

The primary objective of this study was to assess and compare the performance of three popular models used for predicting time-to-event outcomes: the Cox Proportional Hazards model (commonly referred to as Cox Regression), Random Survival Forest, and DeepSurv (a non-linear version of Cox Regression using deep learning techniques). These models were chosen because they represent different approaches commonly employed in survival analysis: Cox Regression as a statistical model, Random Survival Forest as a machine learning model, and DeepSurv as a deep learning model. The aim was tackled via three objectives: 1) compare the models’ performance according to varying sample sizes, 2) compare the models’ performance when the survival distribution deviates from an exponential distribution, and 3) compare the models’ performance under heterogeneity (unobserved heterogeneity when some variables are missing and observed heterogeneity).

## Materials and methods

### Alzheimer data

In our research, we performed a simulation study inspired by clinical Alzheimer’s data from the National Alzheimer’s Coordinating Center (NACC). The actual dataset provided by NACC contains 748 clinical variables. They include age, gender, education, presence/absence of dementia, normal cognitive behavior, patient medical history as well as family history, and many others.

In order to make our simulation study numerically transparent, we first chose a subset of features (variables) from the full set of 748. It is likely that the 748 features have redundancy, thus feature selection would need to be incorporated in the simulations. However, the feature selection was not a part of our three objectives. We considered the 15 identified features from Lin et al. (2018) [5] and then we applied on them a forward selection procedure using a Cox model, which yielded a set of ten features (see S1 Table in S1 File for definitions). Briefly, the selected ten features are

- Demographics information: age (AGE), sex (SEX), years of education (EDUCATION),
- Participant or co-participant reported behavioral information: memory impairment (MEMORY), judgment and problem-solving impairment (JUDGMENT) and difficulty with travelling (TRAVEL),
- Clinical judgment of symptoms: decline in non-memory cognitive abilities (DECCLIN), motor function difficulties (MOTREM), impaired cognitive status (COGSTAT), and decline in memory and cognitive status (DECIN).

For more information, see S1 Table in S1 File.

### Framework followed in our simulation study

Although simulation studies are widely used and come with various advantages [10], they are still lacking proper analysis, design, and report. This fact was pointed out by Morris et al. [21] who therefore introduced new guidelines for simulation studies called ADEMP. The ADEMP recommends structuring a simulation study and reporting five key components: Aims of simulations, Data-generating mechanism, Estimands, Methods, and Performance measures. In what follows, we explain the details of the simulation based on the ADEMP approach.

### Aim of simulations

The aim of our simulation study is to compare the three most common algorithms in the task of predicting the time to MCI progression. We explained the three algorithms in previous sections: Cox proportional hazards model, Random Survival Forests, and DeepSurv.

### Data-generating mechanism

The data-generating mechanism is inspired by a real-world and popular dataset called NACC. We consider one main data-generating algorithm which we then modify into several scenarios. It is crucial that the simulated data mimic a real-life scenario [10], hence here the main algorithm is inspired by the real dataset extracted from NACC in September 2020.

First, we generated the features (predictors) of *N* people, using the following distributions:

$$\begin{array}{c} \begin{array}{c} SEX∼Bernoulli(p_{0}=0.5,p_{1}=0.5)\\ AGE∼Uniform(55,\;85)\\ EDUCATION∼Uniform(0,\;36)\\ MEMORY∼Bernoulli(p_{0}=0.917,p_{1}=0.083)\\ JUDGMENT∼Bernoulli(p_{0}=0.96,p_{1}=0.04)\\ DECCLIN∼Bernoulli(p_{0}=0.95,p_{1}=0.05)\\ TRAVEL∼Multinomial(p_{0}=0.95,p_{1}=0.03,p_{2}=0.02)\\ MOTREM∼Bernoulli(p_{0}=0.99,p_{1}=0.01)\\ COGSTAT∼Multinomial(p_{0}=0.7,p_{1}=0.16,p_{9}=0.14)\\ DECIN∼Bernoulli(p_{0}=0.88,p_{1}=0.12) \end{array} \end{array}$$ (1)

Next, we generated the time of survival for each simulated person. As one of the survival time distributions we used the exponential distribution:

$$\begin{array}{c} T_{exp}=-\frac{log(U)}{\lambda_{i}} \end{array}$$ (2)

with *U* following uniform distribution *U* ∼ *Uni*(0, 1) and $\lambda_{i}=exp\left(X_{i}\hat{\beta }\right)$.

Next, we generated the censoring time for each simulated person. The censoring times were generated following the truncated normal distribution

$$\begin{array}{c} T_{censoring}∼max\left\{0,N\left(\mu =2,\;\sigma =3.611\right)\right\} \end{array}$$ (3)

This lead to the the simulated pair of survival value {*T*, *C*} for each person, where *T* is survival time and *C* is censoring

$$\begin{array}{c} \begin{array}{c} T=min(T_{exp},\;T_{censoring})\\ C=1\;if\;T_{exp}>T_{censoring},C=0\;otherwise \end{array} \end{array}$$ (4)

**To tackle the Objective 1, on sample size** we considered several values of sample size *N*: 250, 500, 2,000 and 6,000. The choice of small sizes 250 and 500 was motivated by the observation that statistical models have higher accuracy than machine learning approaches (including deep learning) in small sample sizes, owing to the fact that the statistical models have a much smaller number of weights (parameters) to train. However, this only applies if the data meet the assumptions of the statistical model.

**To tackle Objective 2, on survival distribution** we additionally simulated the survival times from Weibull and Log-Logistic distributions

$$\begin{array}{c} T_{weibull}={\left(-\frac{log(U)}{\lambda_{i}}\right)}^{1/\beta }\;and\;\;\;T_{log-logistic}=\frac{1}{\lambda_{i}}{\left(\frac{U}{1-U}\right)}^{1/\beta } \end{array}$$ (5)

**To tackle Objective 3 on unobserved heterogeneity,** we also considered scenarios where some features are missing in the analysis. This mimics the situation when some features are either not collected on real patients and then not accounted for in analyses, or it reflects the situation when features are collected but mistakenly not used in the analysis by the prediction models.

**To tackle Objective 3 on observed heterogeneity,** we added heterogeneity into the datasets so we can investigate its effect on the performance of the models. We got inspired by the real NACC dataset where cognitive status (COGSTAT) leads to higher progression risk in males than females (SEX). In the simulations, it was necessary to add dummy features (called interaction variables in statistics) into the dataset. Since (COGSTAT) has three values (0, 1, and 9) we added two dummy features:

$$\begin{array}{c} \begin{array}{cc} f_{COGSTAT,1} & =SEX*I\{COGSTAT==1\}\\ f_{COGSTAT,9} & =SEX*I\{COGSTAT==9\}, \end{array} \end{array}$$ (6)

where *I* is an indicator function. Thus a female with cognitive status 1 (i.e., abnormal test score, coded as *COGSTAT* = 1) gets *f*_*COGSTAT*,1_ = 1, while every other person gets *f*_*COGSTAT*,1_ = 0. Every male gets value 0 for *f*_*COGSTAT*,1_. Also every woman with the cognitive status 0 or 9 gets *f*_*COGSTAT*,1_ = 0. Then in the simulations, this dummy feature will get its own weight. If the weight is positive (negative) then a female with the cognitive status 1 will have a higher (lower) progression risk than a male who has the same cognitive status.

Analogically, a female with cognitive status 9 (i.e., clinician unable to render an opinion, coded as *COGSTAT* = 9) gets *f*_*COGST AT*,9_ = 1, while every other person gets *f*_*COGST AT*,1_ = 0. Every male gets value 0 for *f*_*COGST AT*,9_. Also every woman with the cognitive status 0 or 9 gets *f*_*COGST AT*,9_ = 0. Then in the simulations, this dummy feature will get its own weight. If the weight is positive (negative) then a female with the cognitive status 9 will have a higher (lower) progression risk than a male who has the same cognitive status. The weights (*β* values) for the dummy features are estimated from the real NACC dataset, as explained in the Results section.

### Estimands

Estimands, according to ADEMP guidelines, are the parameters of the data-generating mechanism that are relevant for the predictive algorithms comparisons. In the case of predictive algorithms, a relevant estimand needs to be a measure of prognostic ability. We chose estimands: C-index and Integrated Brier Score which are commonly used for evaluating time-to-event data and are suitable for censored data as well.

### Methods

Methods, according to ADEMP guidelines, are the methods being compared head-on-head in the simulated datasets. In our study they Cox regression, Random survival forests, and DeepSurv (a deep feed-forward neural network).

These methods were chosen based on a recent comprehensive review of Smith et al. [10], which showed the three methods to be the most commonly used in a wide range of medical areas. The review also concluded that it was not quite clear how the three methods compare in terms of accuracy due to the lack of head-on-head comparisons and the lack of studies utilising simulated datasets. In our study, we compare the three methods via simulated datasets.

In the choice of the three methods we were also inspired by the effort to encompass a spectrum of algorithms from three areas of learning from data: statistical learning, machine learning and deep learning. Such choice allows investigating the requirement of the size of training data since such requirement can depend on the number of parameters that need to be tuned. In this respect, the chosen methods differ in the number of parameters to tune, with the smallest number of parameters in statistical learning methods, and the largest number of parameters for deep learning methods.

All three chosen methods are designed to predict the time of the event from survival data. They are able to tackle the challenges of censored data. They allow the inclusion of multiple risk factors (features), non-linear effects and interactions and missing data—however, this is accomplished via different approaches.

The first chosen model is the Cox Proportional Hazard Regression model (Cox model). It belongs to statistical learning approaches. It is the most frequently used statistical learning method for the prediction of the timing of future events from survival data. The missing data imputation, the inclusion of nonlinear effects and the inclusion of interactions need to be engineered by the data analyst. Cox regression has an easy interpretation due to simplicity, and thus, Cox regression is transparent [22]. It is however still unclear if the simplicity always leads to a lower accuracy when compared to machine learning or deep learning methods [10]. This is one of the reasons we study Cox regression here.

The second chosen model is a Random survival forest. It is a machine learning algorithm designed for predicting the times of events from survival data. Its reported strengths lie in its ability to adapt to nonlinearities and interactions in datasets without the need to rely on the data analyst. The advantage is that data analyst does not need to craft them, but the disadvantage is that it is difficult to see what nonlinearities or interactions have been found; thus, it is referred to as a “black box” model. Another advantage is its reasonable computational speed [23]. Our study plans to bring light into the accuracy and the need for training data of a Random survival forest.

The third chosen model is DeepSurv. It is a deep learning model, and it is known as a Cox proportional hazards deep neural network and state-of-the-art survival method for predicting the times of events from survival data [9]. DeepSurv can capture intricate nonlinear relationships as well as interactions, effectively, without relying on the data analyst. However, it is difficult to see how the model works, and thus, it is referred to as a “black box” model. Furthermore, large deep-learning models such as DeepSurv typically require substantial amounts of data to perform well and avoid overfitting. We use the DeepSurv architecture from Katzman [9] we plan to shed light on how much data it needs for the training.

#### Cox proportional hazards model

In survival analysis, the Cox proportional hazards regression model is the most popular statistical predictive model. It is a semiparametric model which does not rely on as many assumptions as parametric models, and therefore, it is widely used [7]. The essential assumption in this model is the proportionality of the hazard function which is defined as:

$$\begin{array}{c} h\left(t,X\right)=h_{0}\left(t\right)e^{\sum_{i=1}^{k}\beta_{i}X_{i}} \end{array}$$ (7)

where *h*_0_(*t*) is the baseline hazard function, *X*_*i*_ represents a vector of predictor variables (features) measured on subject *i*, and *β* is a *k* x 1 vector of unknown parameters (weights). Another assumption of the proportional hazards (PH) models is that the models assume that the hazard ratio (HR) of two people is independent of time i.e. that the proportionality of hazards stays the same over time, hence the name proportional hazard [3, 22, 24]. The model can be built by using partial likelihood and by maximizing the partial likelihood function, the estimates for parameters can be obtained. The partial likelihood can be written as:

$$\begin{array}{c} L\left(\beta \right)=\prod_{i=1}^{j}\frac{e^{\sum_{i=1}^{k}\beta_{i}X_{i}}}{\sum_{l\in R(t_{i})}e^{\sum_{i=1}^{k}\beta_{i}X_{i}}} \end{array}$$ (8)

which is a product of several likelihoods where *R*(*t*_*i*_) is failure time also denoted as the “risk set” of the set of individuals who are at risk at a given time *t*_*i*_ [3, 22].

#### Random Survival Forests

Random survival forest (RSF) is a machine learning method based on extended Breiman’s [25] random forests (RF) method, for time-to-event data which is able to take censoring into account. The process of creating survival trees is similar to it would be for classification or regression. It is based on the bootstrapping and the splitting rule. The bootstrap sample is used to create a tree where randomly selected predictor variables are used to split the root node into two daughter nodes. The splitting rule used in RSF is the log-rank test which tests the dissimilarity of two groups for chosen predictor *x* at split value *c* [26, 27].

The value of the log-rank test is calculated by the following formula:

$$\begin{array}{c} L\left(x,c\right)=\frac{\sum_{i=1}^{N}(d_{i,1}-Y_{i,1}\frac{d_{i}}{Y_{i}})}{\sqrt{\sum_{i=1}^{N}\frac{Y_{i,1}}{Y_{i}}(1-\frac{Y_{i,1}}{Y_{i}})(\frac{Y_{i}-d_{i}}{Y_{i}-1})d_{i}}} \end{array}$$ (9)

where *N* is the number of individuals in node, *d*_*i*,*j*_ is the number of events in the daughter node *j* at time *t*_*i*_ and *Y*_*i*,*j*_ is the number of individuals at risk in the daughter node *j* at time *t*_*i*_. In order to maximize the value of |*L*(*x*, *c*)| which gives us the difference between two groups, we try to find the predictor vector x* and the split value c* that satisfy |*L*(x*, c*)|≥|*L*(*x*, *c*)| [22, 27].

#### DeepSurv

**Fig 1:**
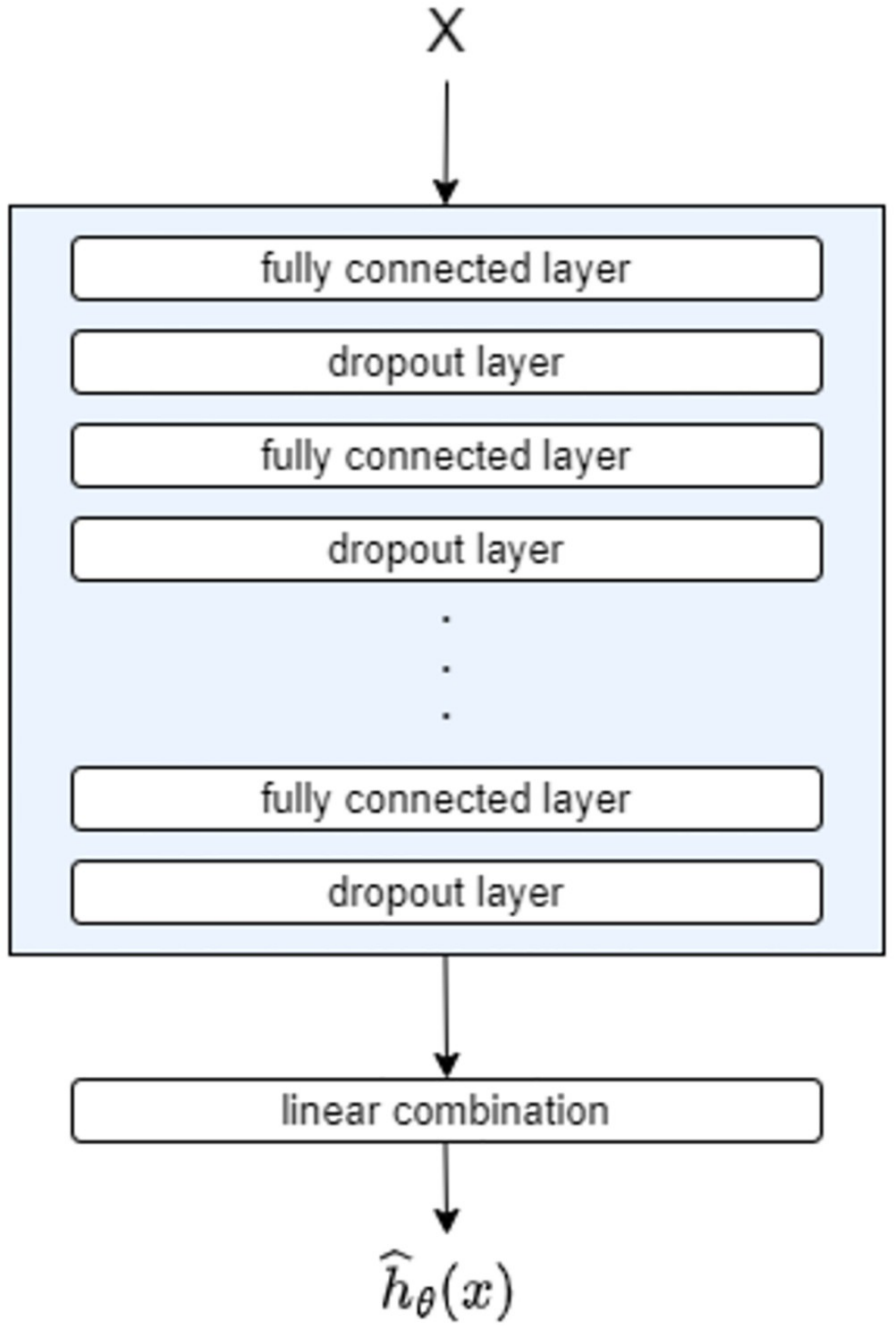
Architecture of DeepSurv model used in this study. The architecture is the same as in [9].

Artificial neural networks have come to the fore in survival analysis. Katzman et al. [9] presented DeepSurv as a non-linear Cox regression model with the use of neural networks. DeepSurv is a deep feed-forward neural network that estimates each individual’s effect on their hazard rates [9]. A diagram of DeepSurv is shown in Fig 1 where **X** is a set of observed predictors (features) as input data. This model is built from hidden layers which are fully-connected nonlinear activation layers followed by dropout layers. The output layer is a single node with a linear activation function. This output is the estimation of the log-risk function denoted as ${\hat{h}}_{\theta }\left(x\right)$. The loss function used in this model is the average negative log-partial likelihood with regularisation [9, 28].

### Performance measures

Performance measures (or evaluation metrics), according to ADEMP guidelines, are the numerical quantities to assess the performance of a method of the algorithm. We chose three performance measures: the mean, 5% lower quartile, and 95% upper quartile. The mean helps to explore the central tendency of the estimated values of the Integrated Brier score and C-index. The lower and upper 5% quartiles help to explore the variability of the Brier score and, hence the algorithm’s uncertainty.

#### C-index

There are multiple metrics available for the evaluation of survival models when taking time-to-event censored data into account. The concordance index (C-index) is one of the most commonly used metrics while multiple types of estimators of the C-index were proposed. Harrell’s C-index [29] is given by formula:

$$\begin{array}{c} C=\frac{\sum_{i,j}I\left({\tilde{T}}_{i}>{\tilde{T}}_{j}\right)\cdot I\left(\eta_{i}>\eta_{j}\right)\cdot \delta_{j}}{\sum_{i,j}I\left({\tilde{T}}_{i}>{\tilde{T}}_{j}\right)\cdot \delta_{j}} \end{array}$$ (10)

and represent the ability of the model to provide reliable survival times based on the individual risk scores. In general, C-index can be understood as the area under the time-dependent ROC curve. Similarly to the AUC if C-index = 1, it represents the best prediction of the model, whereas a C-index of 0.5 corresponds to a random prediction [28, 30].

#### Brier score and integrated Brier score

Another widely used metric to evaluate the prediction accuracy is the Brier score (BS), which represents the average squared distances between the observed survival status and the predicted survival probability [31]. The value of the Brier score lies between 0 and 1 and can be calculated as:

$$\begin{array}{c} BS\left(t\right)=\frac{1}{N}\sum_{i=1}^{N}{\left(1_{T_{i}>t}-\hat{S}\left(t,\vec{x_{i}}\right)\right)}^{2} \end{array}$$ (11)

with perfect accuracy when Brier score = 0. However, the given formula for BS can be used only when there is no censoring present. In the case of right-censored data being present in the dataset, the score needs to be adjusted by weighting the squared distance using the inverse probability of censoring weights method. Thus the formula is such that:

$$\begin{array}{c} BS\left(t\right)=\frac{1}{N}\sum_{i=1}^{N}(\frac{{\left(0-\hat{S}\left(t,\vec{x_{i}}\right)\right)}^{2}\cdot 1_{T_{i}\leq t,\delta_{i}=1}}{\hat{G}\left({\tilde{T}}_{i}\right)}+\frac{{\left(1-\hat{S}\left(t,\vec{x_{i}}\right)\right)}^{2}\cdot 1_{T_{i}>t}}{\hat{G}\left(t\right)}) \end{array}$$ (12)

where $\hat{G}\left(t\right)$ is the estimator of the conditional survival function [28, 31].

The integrated Brier Score (IBS) is an overall measure of model performance at all available times which is calculated with the formula:

$$\begin{array}{c} IBS\left(t_{max}\right)=\frac{1}{t_{max}}\int_{0}^{t_{max}}BS\left(t\right)dt \end{array}$$ (13)

In order to get a good view of C-index and IBS, each generated dataset of sample size *N* was partitioned into a training and testing set, where the training set was used to train a model and the testing set was used to obtain the estimates of C-index and IBS. The most common split ratio was used which is 80:20, where 80% is a training set and 20% is a testing dataset. For each simulation scenario, we simulated the dataset 50 times, thus getting 50 estimates of the C-index and IBS. Then we provide:

- mean C-index and IBS to report the accuracy, and
- 90% confidence interval for C-index and IBS to report the precision. This tells us how much the estimates of C-index and IBS vary from one sample to another, with 90% confidence.

## Results

### Comparison of prediction models at various sample sizes

As planned, in Objective 1 we compare the prediction models at various sample sizes. We consider four scenarios:

*Scenario: Very small sample size*, *N* = 250

*Scenario: Small sample size*, *N* = 500

*Scenario: Medium sample size*, *N* = 2, 000

*Scenario: Large sample size*, *N* = 6, 000

The mean of C-index and IBS is improving with increasing sample size (Table 1, for each predictive model. The largest improvement can be seen in the RSF model where the C-index increased from 60.2% to 72.2%. The DeepSurv has the best improvement in IBS where the score decreased from 14.3% to 7.7%. The CoxPH model showed only small differences in performance when increasing the sample size. In small sample sizes (N = 200 and N = 500), the Cox model achieves the best results (for C-index) (Table 1). At the largest sample size (N = 6,000) the three methods have similar performance. Any differences are small. DeepSurv model is only 0.1% and 0.86% better than CoxPH in terms of C-index and IBS. The distributions of the C-index and Brier score are roughly symmetric (see S1-S4 Figs in S1 File, top rows).

**Table 1:** Comparison of prediction methods according to different sample sizes (N). The mean value and the 90% confidence interval are reported here, over 50 independent simulation runs.

Sample size, N	C-index			IBS		
	CoxPH	RSF	DeepSurv	CoxPH	RSF	DeepSurv
250	0.713 (0.642, 0.762)	0.602 (0.522, 0.687)	0.684 (0.617, 0.752)	0.133 (0.086, 0.174)	0.117 (0.078, 0.168)	0.143 (0.087, 0.202)
500	0.724 (0.681, 0.764)	0.668 (0.622, 0.707)	0.702 (0.654, 0.744)	0.125 (0.090, 0.165)	0.106 (0.071, 0.134)	0.114 (0.087, 0.144)
2,000	0.731 (0.710, 0.754)	0.712 (0.690, 0.738)	0.726 (0.705, 0.744)	0.111 (0.089, 0.129)	0.09 (0.076, 0.109)	0.087 (0.068, 0.105)
6,000	0.730 (0.715, 0.745)	0.722 (0.707, 0.737)	0.731 (0.718, 0.745)	0.103 (0.086, 0.119)	0.083 (0.075, 0.094)	0.077 (0.065, 0.091)

The pairwise differences in C-index show that CoxPH was always better than RSF (see S1-S4 Figs in S1 File, bottom rows).

The precision of C-index and IBS estimates is also improving with increasing sample size. This can be seen in the confidence intervals getting narrower as the sample size increases. Having a narrow confidence interval is desirable, as it means that the estimate (C-index or IBS) does not vary much between samples. At the small sample size (N = 250) the highest precision of C-index and IBS is for the CoxPH model. At the largest sample size (N = 6,000) all models have similar precision of C-index and IBS estimates.

### Comparison of prediction models when simulating data from different survival distributions

Here we compare the performance of the models again, on simulated data from different survival distributions, as planned in Objective 2. We consider three scenarios:

**Scenario: Exponential distribution**. The exponential distribution is the distribution assumed by the CoxPH model. While RSF and DeepSurv do not assume any specific survival distribution.

**Scenario: Weibull distribution**. This distribution was chosen to see how CoxPH will perform when there is a deviation from the Exponential distribution. So effectively this investigates if the CoxPH model is robust against a misspecification of the survival distribution.

**Scenario: Log-logistic distribution**. This distribution was also chosen to see how CoxPH will perform under misspecification of the survival distribution.

The experiments were performed on sample sizes N = 500 (see Table 2) and N = 250 (see S2 Table in Supporting information).

**Table 2:** Comparison of prediction methods at varying survival distributions of data with sample size N = 500 over 50 simulation runs. The mean value and the 90% confidence interval are also reported in the table.

Survival distribution	C-index			IBS		
	CoxPH	RSF	DeepSurv	CoxPH	RSF	DeepSurv
Exponential	0.724 (0.681, 0.764)	0.668 (0.622, 0.707)	0.702 (0.654, 0.744)	0.125 (0.090, 0.165)	0.106 (0.071, 0.134)	0.114 (0.087, 0.144)
Weibull	0.716 (0.679, 0.766)	0.658 (0.612, 0.720)	0.696 (0.655, 0.745)	0.127 (0.086, 0.188)	0.106 (0.076, 0.151)	0.118 (0.079, 0.175)
Log-Logistic	0.673 (0.615, 0.747)	0.623 (0.564, 0.685)	0.626 (0.568, 0.679)	0.188 (0.136, 0.238)	0.167 (0.125, 0.221)	0.208 (0.161, 0.261)

The mean accuracy is the best for the CoxPH model when the data follow the exponential distribution, as it was expected. This is seen by the largest mean C-index and the smallest mean IBS values.

The mean accuracy of the models decreases when the data deviate from the exponential distribution (Weibull or Log-Logistic). The largest decline can be seen in the Log-Logistic survival distribution. The decline in the accuracy is in the CoxPH model which is expected. However, there is a decline in the mean accuracy in models RSF and DeepSurv too.

### Comparison of prediction models in unobserved heterogeneity

In our third objective, we focus on the impact of incomplete information in datasets and heterogeneity. By incomplete information, we mean that the informative variable is not available (e.g. was not collected) for various reasons. This incompleteness was achieved by removing some variables from the simulated dataset and then training the models on fewer features. We consider three scenarios (Table 3):

**Scenario: None unobserved heterogeneity.** Here there is no heterogeneity. This scenario is serving as a baseline scenario to be compared with another two scenarios.

**Scenario: Missing data on cognitive status.** Here the feature COGSTAT is simulated, but then not included in the training set.

**Scenario: Missing data on TRAVEL and DECIN.** Here the features TRAVEL and DECIN are simulated and thus used to derive the survival times, but then they are not included in the training set.

**Table 3:** Comparison of prediction models when unobserved heterogeneity. In each of the three scenarios the ample size N is 2,000 and we used 50 simulation runs to estimate C-index and IBS. The mean value and the 90% confidence interval are also reported in the table.

Heterogenity unobserved	C-index			IBS		
	CoxPH	RSF	DeepSurv	CoxPH	RSF	DeepSurv
None heterogeneity	0.731 (0.710, 0.754)	0.712 (0.690, 0.738)	0.726 (0.705, 0.744)	0.111 (0.089, 0.129)	0.090 (0.076, 0.109)	0.087 (0.068, 0.105)
Missing COGSTAT	0.656 (0.637, 0.675)	0.646 (0.631, 0.660)	0.650 (0.630, 0.670)	0.115 (0.089, 0.142)	0.100 (0.079, 0.123)	0.104 (0.086, 0.128)
Missing TRAVEL and DECIN	0.673 (0.645, 0.702)	0.662 (0.639, 0.690)	0.670 (0.650, 0.693)	0.117 (0.093, 0.145)	0.095 (0.076, 0.117)	0.099 (0.079, 0.125)

It can be seen from the results that the performance of all three models decreased when we removed some information from the simulated dataset. The CoxPH model and DeepSurv model had a similar decrease in accuracy (based on C-index) whilst the RF model had a slightly smaller decrease in accuracy.

When features TRAVEL and DECIN were removed, the decrease in accuracy (C-index) is lower than when we removed COGSTAT. This suggests that COGSTAT has a bigger impact on predicting the conversion to MCI. This could be due to it being an expert test and not a value based on an individual’s own report.

### Comparison of prediction models in observed heterogeneity

In the real NACC dataset, the progression risk related to levels of COGSTAT are different between males and females (SEX), so-called observed heterogeneity. A part of our objective three was to see how the models perform when heterogeneity is present in the dataset. Thus, we simulated three scenarios with different strengths of heterogeneity.

**Scenario: None observed heterogeneity**. Same weights of COGSTAT were used for men and women, calculated from the real NACC dataset:

$$\begin{array}{c} \begin{array}{ccc} male: & \beta_{1}=0.57, & \beta_{9}=-0.44\\ female: & \beta_{1}=0.57, & \beta_{9}=-0.44 \end{array} \end{array}$$ (14)

**Scenario: Small observed heterogeneity**. The same weights of COGSTAT were used for men as before. We changed the weights for women by the mean of the interaction values found in the NACC dataset, which were -0.13 and -0.31 for COGSTAT 1 and 9 respectively. So the weights for the small observed heterogeneity were

$$\begin{array}{c} \begin{array}{ccc} male: & \beta_{1}=0.57, & \beta_{9}=-0.44\\ female: & \beta_{1}=0.57-0.13, & \beta_{9}=-0.44-0.31 \end{array} \end{array}$$ (15)

**Scenario: Medium observed heterogeneity.** Same weights of COGSTAT were used for men as before. We changed the weights for women by the sum of the mean and of one standard deviation of the interaction values. The estimated standard deviations were 0.13 and 0.16, for COGSTAT 1 and 9 respectively from NACC dataset. So the weights for the medium heterogeneity were

$$\begin{array}{c} \begin{array}{ccc} male: & \beta_{1}=0.57, & \beta_{9}=-0.44\\ female: & \beta_{1}=0.57-\left(0.13+0.13\right), & \beta_{9}=-0.44-\left(0.31+0.16\right) \end{array} \end{array}$$ (16)

**Scenario: Large observed heterogeneity**. Same weights of COGSTAT were used for men as before. We changed the weights for women by sum of the mean and of two standard deviations of the interaction values. The estimated standard deviations were 0.13 and 0.16, for COGSTAT 1 and 9 respectively from NACC dataset. So the weights for the medium heterogeneity were

$$\begin{array}{c} \begin{array}{ccc} male: & \beta_{1}=0.57, & \beta_{9}=-0.44\\ female: & \beta_{1}=0.57-\left(0.13+2\times 0.13\right), & \beta_{9}=-0.44-\left(0.31+2\times 0.16\right) \end{array} \end{array}$$ (17)

In the simulated three scenarios of observed heterogeneity, the accuracy of the three models does not change. For example, the CoxPH model has C-index is 73.1% for non-heterogeneity, 72.4% for small, 72.9% for medium, and 73.4% for large heterogeneity (Table 4). The differences are small and can be due to random variation. In the scenarios, none of the models decreases in the accuracy of the prediction, when the heterogeneity was ignored.

**Table 4:** Comparison of prediction methods at various scenarios of observed heterogeneity: None, small, medium, and large. The size of simulated data is *N* = 2, 000, size of training data is 80% of N = 2,000. The mean value and the 90% confidence interval are reported here, over 50 simulation runs.

Observed heterogeneity	C-index			IBS		
	CoxPH	RSF	DeepSurv	CoxPH	RSF	DeepSurv
*None*	0.731 (0.710, 0.754)	0.712 (0.690, 0.738)	0.726 (0.705, 0.744)	0.111 (0.089, 0.129)	0.090 (0.076, 0.109)	0.087 (0.068, 0.105)
*Small*	0.724 (0.707, 0.742)	0.705 (0.684, 0.724)	0.723 (0.704, 0.746)	0.114 (0.090, 0.144)	0.087 (0.068, 0.106)	0.084 (0.067, 0.101)
*Medium*	0.729 (0.703, 0.754)	0.710 (0.681, 0.741)	0.730 (0.702, 0.752)	0.115 (0.094, 0.136)	0.088 (0.073, 0.107)	0.082 (0.064, 0.101)
*Large*	0.734 (0.709, 0.756)	0.718 (0.682, 0.745)	0.733 (0.701, 0.756)	0.115 (0.090, 0.136)	0.082 (0.070, 0.096)	0.078 (0.057, 0.094)

## Discussion

### Contributions

We proposed and implemented a novel simulation framework in Python to simulate time-to-MCI conversion in patients, and then we used it to compare the three most common predictive algorithms for time-to-event data that are subject to the right censoring [10]: one classical statistical predictive algorithm (Cox proportional hazards model, CoxPH), one machine learning algorithm (Random survival forest, RSF) and one deep learning algorithm (DeepSurv). We compared them under various scenarios: varying sample sizes, varying survival distributions of data, incomplete datasets, and heterogeneity of risks for males and females.

In our simulations, inspired by the NACC dataset, the CoxPH prediction algorithm showed the best accuracy compared to the other two algorithms, when the training sample size was small at *n* = 200. The C-index was better by 11.1% (compared to RSF) and by 2.9% (compared to DeepSurv). This was not surprising because, when having a small number of patients for training, the predictive algorithms with fewer training parameters (weights) have better accuracy—as long as the data-generating mechanism is similar to the assumptions of the predictive algorithms. Deep learning algorithms are data-hungry [17, 32], while there is no easy way to do sample size calculation. We found here that the training size of 4800 patients is needed (4800 = 80% of 6000) for the deep learning model to have the same accuracy of up to 0.001 difference in C-index measured on 0–1 scale (so up to 0.1% difference).

When we simulated data from a generating mechanism different from the CoxPH model, in the sense that the survival distribution is not exponential we found that all three algorithms decreased in their prediction accuracy. However, the decrease in model performance depended on differences in how survival distribution was calculated. A bigger deterioration in the accuracy was seen for the Log-Logistic distribution where the C-index reached 7% smaller accuracy in the DeepSurv model than with the Weibull distribution. We did not witness an advantage of DeepSurv or RSF against CoxPh, which is similar to the finding of Austin et al. [18] study.

Next, we investigated scenarios of unobserved heterogeneity, when one or two features were omitted from the training and prediction. This aims to mimic a real-life situation when some feature or variables are not collected for various reasons, or not used for prediction. Our study shows that missing information on cognitive status (COGSTAT) or on travelling difficulty (TRAVEL) and a decline in the subject’s memory (DECIN) can lead to lowered prediction accuracy in all three considered models. Besides that, some predictive variables have a greater influence on model performance than others. This was confirmed by our experiments when removing COGSTAT from data led to worse accuracy in an average of 7.2% among all models but removing only TRAVEL and DECIN resulted in a 5.5% decrease in models performance. Such investigation is novel, as to our knowledge this was not investigated for Alzheimer’s disease. It gives us important knowledge: if our patients are heterogeneous in their risk of MCI progression, and if the relevant features are not measured then all three algorithms will drop in accuracy.

When the simulated data exhibited observed heterogeneity of risks we found that the CoxPH model gave the same accuracy as RSF and DeepSurve—which is a novel result. The heterogeneity of risks is crucial to recognize so that we can choose a predictive algorithm that has the highest accuracy. In the real Alzheimer data from NACC, we found that males and females have significantly different risk estimates for cognitive status (COGSTAT) hence there was a heterogeneity. Specifically, if someone has cognitive status abnormal (COGSTAT = 1) the risk of progression to MCI increases but the amount of increase is higher in men than in women, which is an observed heterogeneity. In the published reports of comparative analysis of predictive algorithms, the heterogeneity of risks was not investigated. Thus our work brings novel contributions to understanding the performance of the chosen three algorithms in case of the observed heterogeneity.

On the relevant note, the existing published reports did investigate the non-linearity of risks (e.g. quadratic), but then they used a linear CoxPH model which not surprisingly showed a decrease in accuracy and deep learning models did not decrease in the accuracy [10]. Here we found that if we use linear Cox PH without heterogeneity, its accuracy drops, and when we use the Cox PH model with heterogeneity, then its accuracy is the same as that of the deep learning method. Such a comparison was never done for predictive algorithms. One reason may be that the Python library does not have the heterogeneity implemented in the Cox PH model, so we had to code it by creating the interaction dummy variables.

Finally, it is important to discuss the evaluation of the predictive algorithms on larger simulated datasets and on real datasets. We found the computer-generated datasets to be adequate for the scope of this study while the largest dataset contained 6,000 patients. When we increased the sample size beyond 6,000, we observed that the accuracy of all models was not improving any further. Regarding the evaluation of the real-world datasets there is a work of Lin et al. [5] who utilised the NACC dataset in conjunction with machine learning techniques to predict the conversion to Mild Cognitive Impairment (MCI) and Alzheimer’s Disease (AD). In their research, they employed ROC AUC as the metric of evaluation and approximately 75% accuracy was achieved. In our study, we achieved slightly lower accuracy (73% for C-index). Thus the models used in our study came very close to matching the level of performance reported by Lin et al. However, it is noteworthy that Lin et al.’s study utilised a much more expansive dataset, encompassing not only a greater number of patients but also a wider array of predictive variables. In future, it will be beneficial to do more real-world data comparisons of the predictive algorithms.

### Advantages of our approach

We did a systematic comparison of one statistical predictive algorithm, one machine learning, and one deep learning algorithm to predict time to MCI conversion. There is a lack of such comparisons mainly because the data-generating mechanism is complex to propose and implement. Some existing papers have only one or two features, we had 10 features that were chosen from the existing trained Cox PH model. Only via computer simulation, we can understand in which data-generating mechanisms do certain predictive algorithms work better than others [10].

The main advantage of our paper is in comparing the predictive algorithms via implementing a computer simulation of time to MCI conversion data sets. The existing data-generating simulations show a lack of plausibility in the sense that they are often biased toward the machine-learning approach. For example, they simulate a non-linear relationship between features and time to conversion and then apply a CoxPH model with linear effects, concluding that a neural network is “better” [10]. Here, we simulated the heterogeneity of risk factor cognitive status (COGSTAT) and then applied a CoxPH model with heterogeneity, to avoid such bias. Namely, in the NACC dataset, the impaired cognitive status (COGSTAT) leads to higher progression risk in males than females (SEX). We manually inserted the heterogeneity weights into the CoxPH model, since the Python library for CoxPH does not have heterogeneity implemented as an option.

The equal performance of the CoxPH, RSF, and DeepSurve algorithms is an advantage because it suggests that statistical models are capable of solving problems similar to those that can be solved by machine learning algorithms. Statistical models are often more interpretable and transparent than more complex machine learning models, which are based on implicit relationships learned from data. The results of statistical models can often be explained in terms of assumptions of the model, making it easier to understand the relationships between features (predictors).

Explainable and interpretable models that are also highly accurate are often preferred in the medical domain. This is signified by EU regulations requiring such methods to be transparent etc (can reference this). The benefit of simulation studies like ours is thus of high benefit, yet infrequently done. As we illustrated, in a simulation study we can see when and how which model works better and we can then consider the best use-case scenario for the model. I.e. if via simulation DeepSurv and CoxPH models give equivalent performance then in the medical application where data is often limited and models require transparency the CoxPH would be the favored approach.

We created a data-generating mechanism and implemented it in Python, so this can be used by others in the future to investigate the comparisons further and to do sample size calculations too. An additional benefit is the detailed description of the simulation study, which the authors [10] have criticized in other articles for their lack of important information in their reports.

### Disadvantages of our approach and what can be done in future

A limitation of our study is in our data generating mechanism because we did an independent simulation of feature values i.e. we assume independence of the features. In the future, this can be further expanded by using conditional distributions.

In our simulated data, we did assume no outliers. Indeed, in real medical data, it is not uncommon to have unusual observations. It is therefore important, in a future study, to simulate e.g. 5% outliers and study how the models perform in the presence of outliers. If their performance does not decrease much, then they are called to be robust.

Although the implementation of baseline models offers an advantage of aligning with state-of-the-art approaches, it does come with potential limitation. In future, we encourage to explore the modifications or customizations of these models, e.g. modifying layers and parameters in deep learning model, to enhance their performance as well as their adaptability to specific medical domains.

## Conclusion

We showed how to do computer simulations in a novel medical domain of progression to MCI in Alzheimer’s disease however this is applicable to any medical domain with survival data. We illustrated that the comparison of predictive algorithms is complex and which algorithm is the best does depend on a combination of several factors such as sample size, survival distributions of data, missing information, or heterogeneity.

As some of the related articles demonstrate, we were able to verify the significance of the sample size of the data used for model training. For machine learning models to perform better or equal to classical statistical algorithms, the training sample size needs to be large. The performance of models is affected by the type of survival distribution. The performance of models is affected by the type of survival distribution of the time-to-event data and the heterogeneity of the risks. When the data have exponential distribution, we found that deep learning predictions and Cox model predictions have the same performance, as long as there is a large enough training dataset for deep learning and as long as heterogeneity is included in the Cox model.

When it comes to missing information in data, it is problematic not only for statistics but also for machine learning methods, because it can cause a significantly worse accuracy in model prediction. Simulation studies are crucial for prediction algorithm comparisons, especially because we can simulate any level of heterogeneity, and thus create a fair comparison of algorithms.

## Supporting information

S1 File (PDF)
