# Supplementary material for: Comparing the performance of statistical, machine learning, and deep learning algorithms to predict time-to-event: A simulation study for conversion to mild cognitive impairment
Source: PLoS One. 2024 Jan 22;19(1):e0297190. doi: 10.1371/journal.pone.0297190 (PMC10802955; doi:10.1371/journal.pone.0297190)
Supplement: S1 File — (PDF) [file pone.0297190.s001.pdf]

Supporting information

S1 Table. Description of ten features from NACC dataset

Variable name	Form	Description	Levels
SEX	Demographics	Subject's sex	0 = male<br>1 = female
AGE	Demographics	Subject's age	–
EDUC	Demographics	Years of education	–
MEMORY	CDR® Plus<br>NACC FTLD	Memory	0 = no impairment<br>1 = some impairment
JUDGMENT	CDR® Plus<br>NACC FTLD	Judgment and problem-solving	0 = no impairment<br>1 = some impairment
DECCLIN	Clinician Judgment of Symptoms	Clinician believes there is a meaningful decline in memory, non-memory cognitive abilities, behavior, ability to manage his/her affairs, or there are motor/movement changes.	0 = no<br>1 = yes
TRAVEL	Functional Activities Questionnaire	In the past four weeks, did the subject have any difficulty or need help with: Traveling out of the neighborhood, driving, or arranging to take public transportation.	0 = normal<br>1 = has difficulty but does by self<br>2 = requires assistance
MOTREM	Clinician Judgment of Symptoms	Indicate whether the subject currently has meaningful changes in motor function - Tremor	0 = no<br>1 = yes
COGSTAT	Neuropsychological Battery Summary Scores	Per clinician, based on the neuropsychological examination, the subject's cognitive status is deemed.	0 = normal or better than normal for age<br>1 = abnormal test scores<br>9 = clinician unable to render opinion
DECIN	Clinician Judgment of Symptoms	Does the co-participant report a decline in subject's memory (relative to previously attained abilities)?	0 = no<br>1 = yes

S1 Fig. Comparison of the prediction models at sample size $N = 250$. Top row: the boxplots of the 50 values of the C-index and Brier score. Bottom row: the boxplots of pairwise differences between the prediction models. For example, the first boxplot shows differences in the C-index of the CoxPH minus RSF model, thus a positive difference means that CoxPH is a better model than RSF in the simulated data.

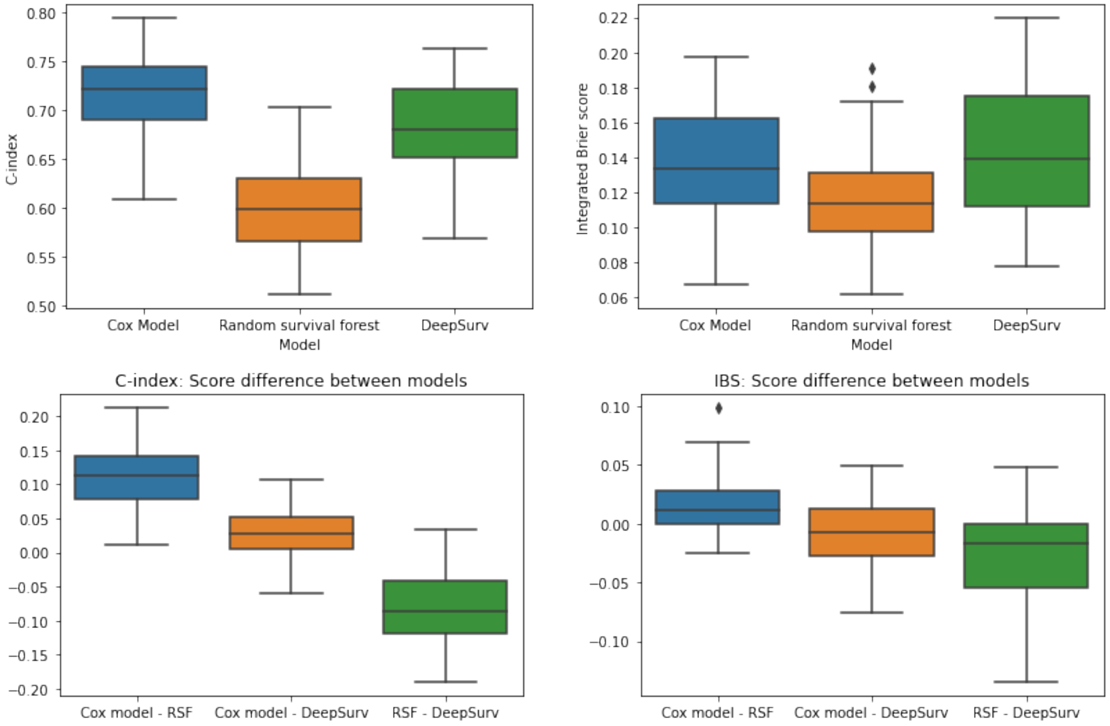

S1 Fig.

S2 Fig. Comparison of the prediction models at sample size $N = 500$. Top row: the boxplots of the 50 values of the C-index and Brier score. Bottom row: the boxplots of pairwise differences between the prediction models. For example, the first boxplot shows differences in the C-index of the CoxPH minus RSF model, thus a positive difference means that CoxPH is a better model than RSF in the simulated data.

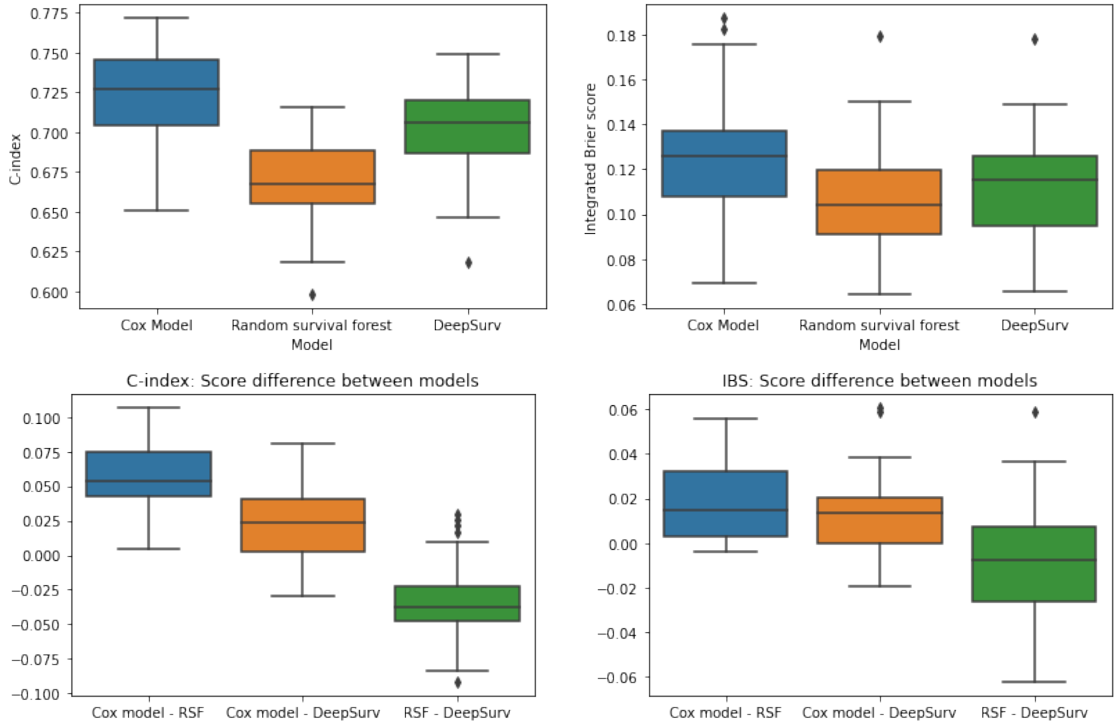

S2 Fig.

S3 Fig. Comparison of prediction models at sample size $N = 2,000$. Top row: the boxplots of the 50 values of the C-index and Brier score. Bottom row: the boxplots of pairwise differences between the prediction models. For example, the first boxplot shows differences in the C-index of the CoxPH minus RSF model, thus a positive difference means that CoxPH is a better model than RSF in the simulated data.

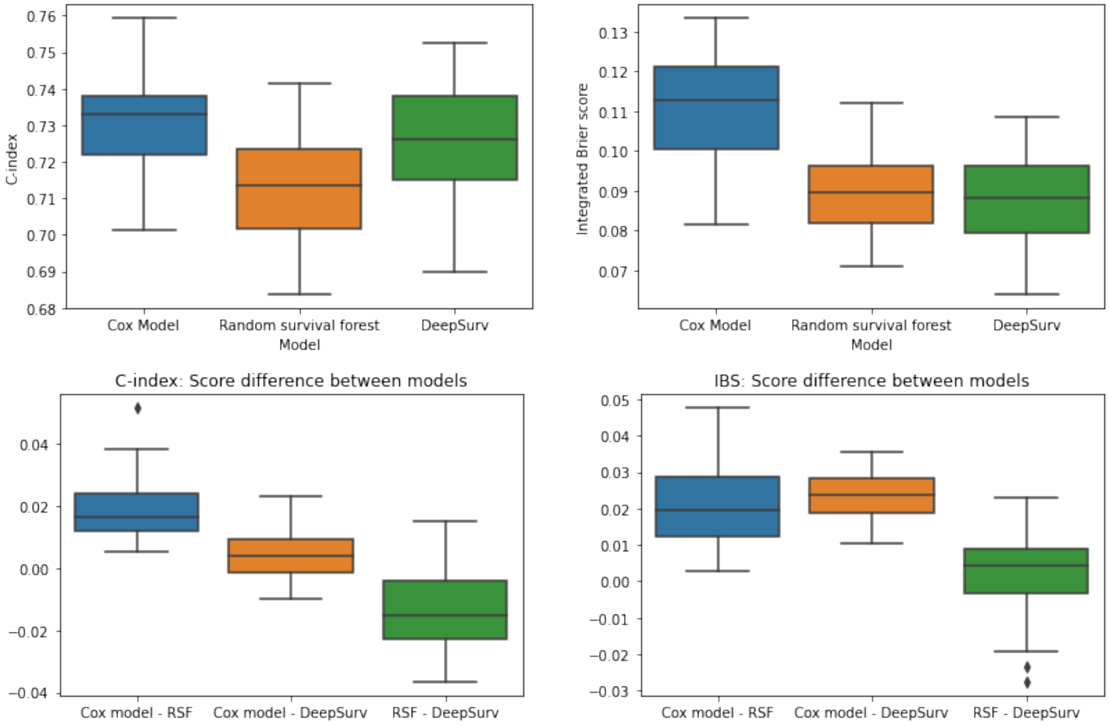

S3 Fig.

S4 Fig. Comparison of prediction models at sample size $N = 6,000$. Top row: the boxplots of the 50 values of the C-index and Brier score. Bottom row: the boxplots of pairwise differences between the prediction models. For example, the first boxplot shows differences in the C-index of the CoxPH minus RSF model, thus a positive difference means that CoxPH is a better model than RSF in the simulated data.

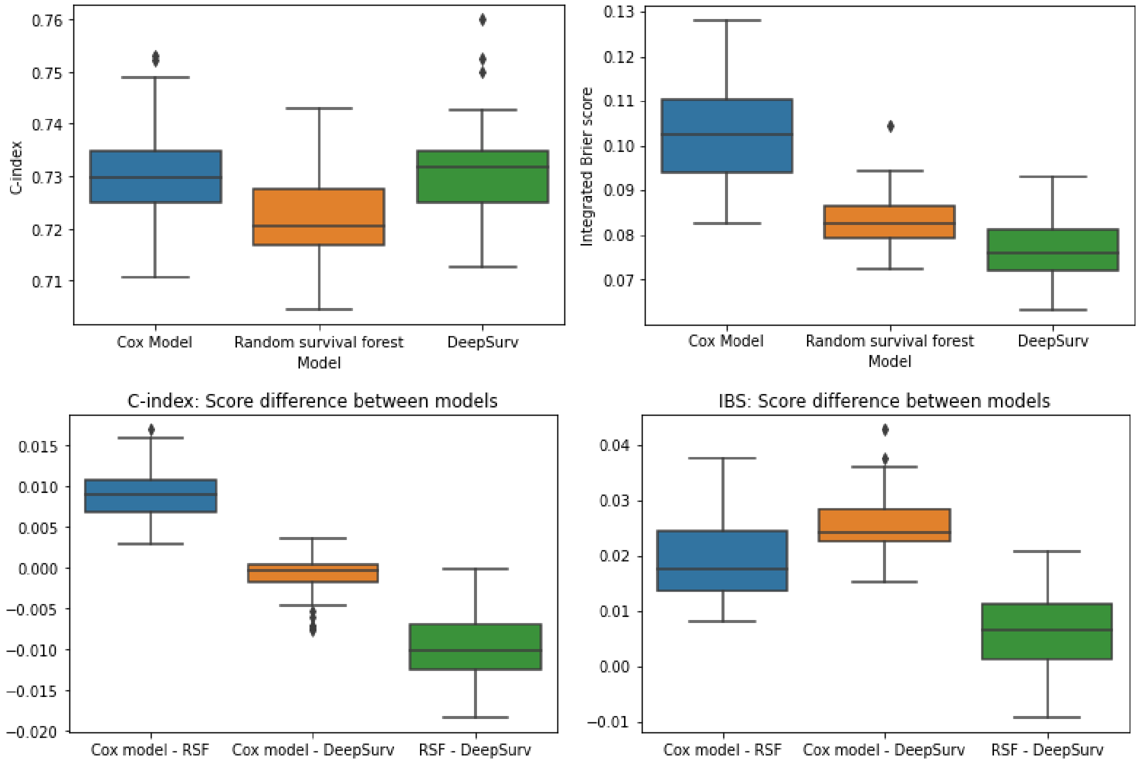

S4 Fig.

S2 Table. Comparison of prediction methods according to varying survival distributions with sample size $N = 250$ over 50 simulation runs. The mean value and the 90% confidence interval are reported in the table.

Survival distribution	C-index			IBS		
	CoxPH	RSF	DeepSurv	CoxPH	RSF	DeepSurv
Exponential	0.713<br>(0.642, 0.762)	0.602<br>(0.522 , 0.687)	0.684<br>(0.617, 0.752)	0.133<br>(0.086, 0.174)	0.117<br>(0.078, 0.168)	0.143<br>(0.087, 0.202)
Weibull	0.721<br>(0.614, 0.787)	0.606<br>(0.518, 0.690)	0.683<br>(0.605, 0.770)	0.132<br>(0.086, 0.177)	0.116<br>(0.086, 0.145)	0.141<br>(0.080, 0.196)
Log-Logistic	0.670<br>(0.599, 0.755)	0.573<br>(0.514, 0.639)	0.613<br>(0.509, 0.708)	0.191<br>(0.147, 0.251)	0.177<br>(0.123, 0.250)	0.246<br>(0.153, 0.353)
